# Seroepidemiological survey of contagious bovine pleuropneumonia among cattle in El Jazeera State (Central Sudan)

**DOI:** 10.1186/s13620-024-00271-2

**Published:** 2024-05-17

**Authors:** Mohammed Hussien, Eslah Abdelhabib, Abdalsalam Hamid, Azza Musa, Huyam Fadolelgaleel, Shima Alfaki, Abdel Rahim El Hussein

**Affiliations:** 1 Central laboratory, Ministry of Higher Education and Scientific Research, P.O. Box 7099, Khartoum, Sudan; 2 Central Veterinary Research Laboratory (CVRL), Animal Resources Research Corporation (ARRC), El Amarat, P.O. Box 8067, Khartoum, Sudan; 3 Department of Microbiology, Faculty of Veterinary Medicine, University of Khartoum, Khartoum North, P.O. Box: 32, Khartoum, Sudan

**Keywords:** CBPP, Cattle, cELISA, Seroepidemiology, Survey, Sudan

## Abstract

**Background:**

Contagious bovine pleuropneumonia (CBPP) is an economically important infectious disease that is characterized by a variable course and insidious nature. A cross-sectional study was conducted in El Jazeera State, Central Sudan, to determine the seroprevalence and risk factors of CBPP in cattle from seven localities. A total of 218 serum samples were randomly collected from apparently healthy cattle aged older than 6 months between April and May 2021 and were tested serologically using a commercial ELISA kit.

**Results:**

The overall seroprevalence of CBPP was 50.5% (110/218). Univariate analysis showed a significant difference (*p* < 0.05) between sex, locality and water source and seropositivity to CBPP. Multivariate analysis revealed that the independent risk factors (sex, locality and water source) were also statistically significant (*p* < 0.05). At herd level, out of 20 herds 16 (80%) proved to be positive for CBPP antibodies. It is apparent from the present study that CBPP infection is prevalent among cattle in El Jazeera State, Central Sudan.

**Conclusions:**

To the best of our knowledge, this is the first seroepidemiological study on CBPP infection in Central Sudan. The authors recommend major awareness both in the production area and quarantine centers, as CBPP may result in restrictions on the international trade of animals and animal products.

**Supplementary Information:**

The online version contains supplementary material available at 10.1186/s13620-024-00271-2.

## Background

Contagious bovine pleuropneumonia (CBPP) is an infectious and highly contagious respiratory disease of cattle in Africa, caused by *Mycoplasma mycoides* subsp. *Mycoides* (*Mmm*) [1]. Available veterinary records have showed that CBPP has been enzootic in Sudan since the beginning of the century. The disease was first observed in Darfur Province in 1875, and later spread to Khartoum Province, where it caused great losses among cattle [2].

Transmission occurs through direct contact between infected and susceptible animals. The main route of infection is airborne via infected droplets [3], which has been confirmed experimentally [4]. The disease has a significant economic impact on cattle owners owing to high mortality rates, loss of production, disease control costs, loss of weight and workability, delayed marketing, decreased fertility, loss due to quarantine, loss of cattle trade, and reduced investment in livestock production [5, 6]. The options for control of CBPP include cattle movement control and quarantine, test and slaughter, antimicrobial treatment, and vaccination with a live vaccine [1]. Most of these measures cannot be implemented in Africa due to absence of compensation mechanisms for the farmers and the antimicrobial treatment does not necessary result in elimination the causative agent [7], while the live vaccines have several shortcomings such as severe side reactions and limited duration of immunity [8]. According to the Terrestrial Animal Health Code [6], serological surveillance of CBPP is more suitable. The complement fixation test (CFT) and competitive enzyme-linked immunosorbent assay (c-ELISA) are serological tests recommended by the OIE for herd-level serological diagnosis and are commonly used for disease investigation in Africa [9]. CFT or c-ELISA can be used to become aware of natural infections in cattle herds in Africa, even in regions where vaccination campaigns have not been orderly carried out, due to the fact put up post-vaccination antibodies do not persist after 3 months [10]. Both techniques are beneficial only in CBPP diagnosis in herds and not in individual infected cattle. c-ELISA using a specific monoclonal antibody targeting *Mmm* antigens has been used in many herds in Africa [11–13]. Cross-reactions with other *Mycoplasma* species have not been recorded, but 96% sensitivity and 97% specificity have been reported [13, 14]. Kebede et al. [15] estimated seroprevalence and assessed the associated risk factors of CBPP using c-ELISA in the Bench-Maji Zone in Southwest Ethiopia.

In Sudan, only a few recent studies have used c-ELISA to evaluate the prevalence, risk factors, and geographical distribution of CBPP to identify the high-risk areas. A cross-sectional study was conducted to determine the impact of CBPP in various pastoral areas of Khartoum State, Sudan. The results showed that 46% of the 300 non-vaccinated samples were positive, while 43% of the 86 vaccinated samples were positive. This requires the implementation of appropriate vaccination programs and control measures to reduce economic losses associated with CBPP [16]. In the Eastern States of Sudan, the highest seroprevalence was observed in Al Gedarif State (12%), followed by Kassala (6.9%), and Red Sea State (4.1%) [17].

Another study on seroprevalence and risk factors of CBPP in various pastoral areas in Khartoum State was conducted to detect the incidence of specific antibodies against *Mmm*, and 386 serum samples were examined. In that study, the overall seroprevalence was 45.3% with regard to risk factors, some of which had a significant association with community knowledge, age, sex, breed, herd structure, herd size, and the number of dead animals [18].

Owing to the meagre data available on CBPP disease in El Jazeera State (Central Sudan), this survey was thus carried out to determine the seroprevalence and risk factors of CBPP in cattle in seven localities in the state.

## Methods

### Study area

**Fig. 1:**
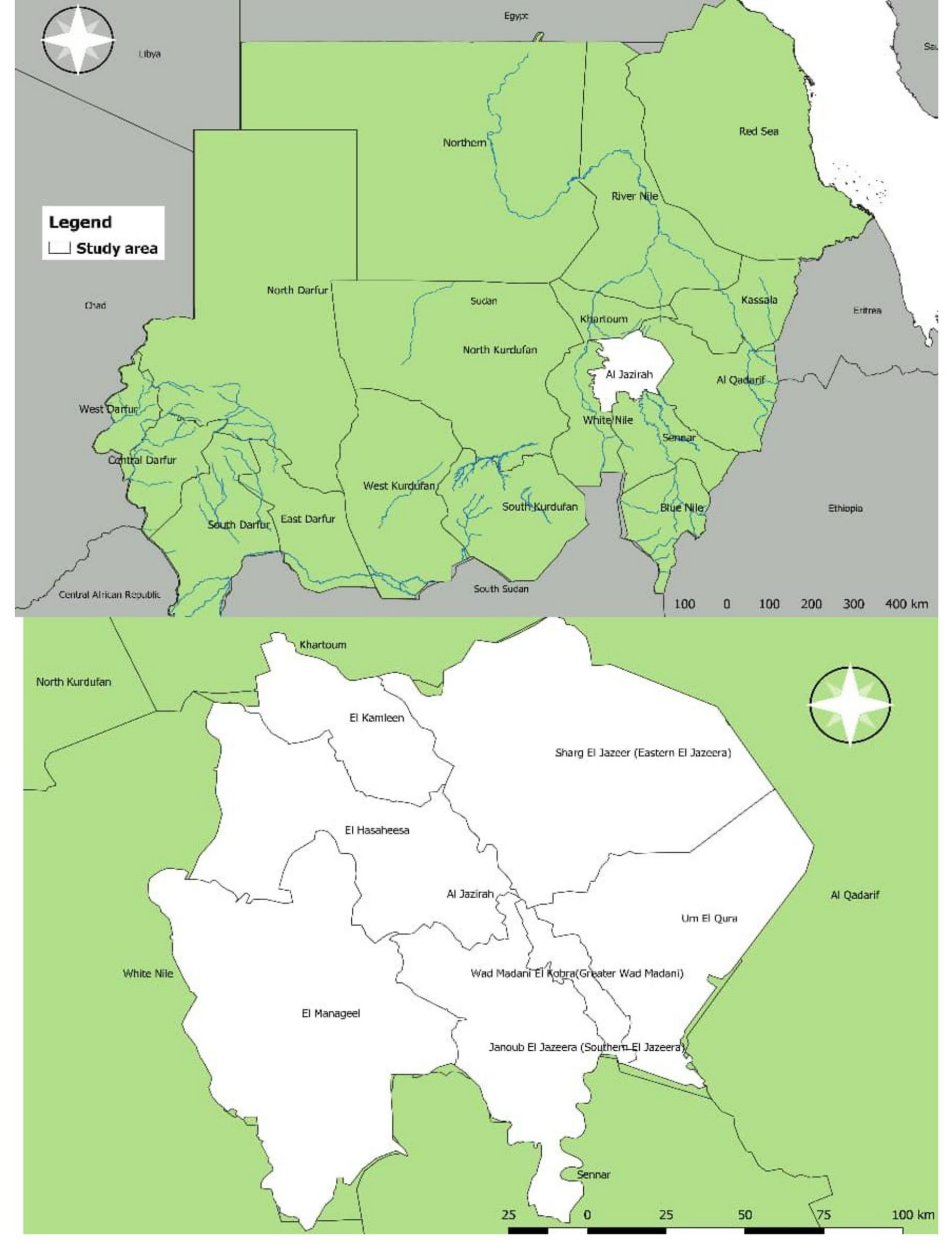
Map of El Jazeera State, Sudan showing the locations where serum samples were collected

This study was carried out in seven localities in El Jazeera State. The state lies between latitudes 14° 13.872’ N and longitudes 33° 21.582’ E, in the central region of the country. It is boarded to the north by Khartoum State, south by Sinnar State, west by White Nile State, and east by Gedarif State (Fig. 1). The climate is dry, with a short rainy season from July to September.

### Study design

A cross-sectional study was conducted to determine the prevalence of antibodies against CBPP in cattle using a competitive enzyme-linked immunosorbent assay (c-ELISA). Sample size for this survey was estimated using the formula n = z^2^PQ/ L^2^ [19], where 𝑛 was the required number of individuals to be examined; z was a constant = 1.96; P is a known or estimated prevalence; Q= (1-P); 𝐿 is the allowable error. The estimated number using this formula assuming the 12% prevalence rate [20] was 162. A total number of 218 serum samples were included in this survey.

### Sample collection

Samples were collected according to the Animal Welfare Code of Sudan. This study was carried out in compliance with STROBE statement. Blood was collected from the animals by qualified veterinarians following proper physical restraint to ensure both personnel and animal safety. A total of 218 blood samples (140 crossbred and 78 indigenous animals) were randomly collected using lottery method from apparently healthy animals aged older than 6 months between April and May 2021. The production system of the cattle sampled is pastoralism. Samples were collected from seven different localities in El Jazeera State, including El Manageel, Wad Madani, Eastern El Jazeera, El Kamleen, El Hasaheesa, Twenty-fourth El Gorashi, and Southern El Jazeera (Fig. 1). A herd was considered positive for CBPP infection when at least one animal with positive antibody reaction was detected. Serum was separated and frozen at -20^o^C until tested. The risk factors analyzed in this study were age, sex, breed, locality, and water source. Data were collected from animals’ owners.

### Serological screening using c-ELISA

The serum samples were examined for antibodies against *Mmm* using c-ELISA (IDEXX, Institute Pourquier, Montpellier, France) according to the manufacturer’s instructions. Optical densities (OD) were estimated using a microplate ELISA reader (Stat Fax 4200, USA) at wavelength of 450 nm. Sera with percentage inhibition (PI) > 50% were considered positive.

### Statistical analyses

The Statistical Package for Social Science (SPSS) software (IBM version 20.0, USA) was used to analyze the results. Logistic regression analysis was used to assess the association between various risk factors and the prevalence of CBPP. A multivariable model for the outcome variable was constructed using logistic regression and the chi-square. A *p*-value of ≤ 0.05 indicated a significant association.

## Results

The ELISA results revealed that the overall seroprevalence of CBPP-specific antibodies was 50.5% (110/218) among apparently healthy cattle in El Jazeera State. The highest prevalence of CBPP seropositivity was recorded in Southern El Jazeera (86.7%), whearas the lowest prevalence was recorded in the Twenty-fourth El Gorashi locality (12.9%) (Table 1). The variation in seroprevalence among the seven localities was statistically significant (*p* = 0.0001). Univariant logistic regression showed a significant difference (*p* < 0.05) between sex, locality, water source and seropositivity to CBPP. The highest seropositivity rate was noted in females (54.9%) compared to males (32.6%). Moreover, the locality showed a significant difference (*p* < 0.05), and the highest seropositivity rate was noted in the Southern El Jazeera (86.7%) locality. The water source of canar showed the highest seropositivity rate (80%) (Table 2). Multivariate analysis revealed that the independent risk factors (sex, locality and water source) were statistically significant (*p* < 0.05). Females were more likely to be infected with CBPP compared to males (*p* = 0.009). Cattle sampled from Eastern and Southern El Jazeera and Wad Madani localities were more likely to be infected (*p* = 0000). Canar as a water source for drinking increased the risk of contracting CBPP compared to other sources of water (*p* = 0.001) (Table 3). Regard to infection at herd level, out of 20 herds 16 (80%) proved to be positive for CBPP antibodies. The highest infection rate (100%) was observed in Southern El Jazeera, Wad Madani and Twenty four Gorashi (Table 4).

**Table 1:** Univariate analysis for the association of origin of collected samples (locality) and seropositivity for CBPP in cattle in El Jazeera State in Sudan during the period between April – May, 2021

Locality	No of tested cattle	No positive	Prevalence rate in cattle (%)	*p*-value
El Manageel	31	11	35.5	0.0001^*^
Wad Madani	30	24	80.0	
Eastern El Jazeera	32	16	50.0	
El Kamleen	32	14	43.8	
El Hasaheesa	32	15	46.9	
Twenty-four El Gorashi	31	4	12.9	
Southern El Jazeera	30	26	86.7	
**Total**	**218**	**110**	**50.5**	

*significantly different, with a *p* < 0.05

**Table 2:** The Univariable association between potential risk factors and CBPP seropositivity among cattle in El Jazeera State, Sudan using the chi-square test

Risk factor	Groups	Animals tested	Animals affected (%)	*p*-value
**Age (years)**	< 3	89	39 (43.8)	0.257
	3–6	54	29 (53.7)	
	> 6	75	42 (56.0)	
**Sex**	Male	43	14 (32.6)	0.009^*^
	Female	175	96 (54.9)	
**Breed**	Cross	140	67 (47.9)	0.303
	Local	78	43 (55.1)	
**Locality**	Southern El Jazeera	30	26 (86.7)	0.0001^*^
	Wad Madani	30	24 (80.0)	
	El Manageel	31	11 (35.5)	
	Twenty-four El Gorashi	31	4 (12.9)	
	El Hasaheesa	32	15 (46.9)	
	El Kamleen	32	14 (43.8)	
	Eastern El Jazeera	32	16 (50.0)	
**Water source**	Barraled	93	53 (57.0)	0.0001^*^
	Canar	30	24 (80.0)	
	Turaa	95	33 (34.7)	

*significantly different, with a *p* < 0.05

Age: x^2^: 2.718 df: 2

Sex: x^2^: 6.866 df: 1

Breed: x^2^: 1.05 df: 1

Locality: x^2^: 47.221 df: 6

Water source: x^2^: 21.453 df: 2

**Table 3:** Multivariate analysis using logistic regression model for significant association (*p* < 0.05) of risk factors and CBPP seropositivity among cattle in El Jazeera State, Sudan

Risk factor	Animals tested	Animals positive (%)	*p*-value	Odds ratio	95% CI Lower - Upper
**Sex**					
Male Female	43 175	14 (32.6) 96 (54.9)	Ref 0.009^*^	- 2.939	- 1.315–6.569
**Locality**					
Twenty-four El Gorashi Southern El Jazeera Wad Madani El Manageel El Hasaheesa El Kamleen Eastern El Jazeera	31 30 30 31 32 32 32	4 (12.9) 26 (86.7) 24 (80.0) 11 (35.5) 15 (46.9) 14 (43.8) 16 (50.0)	Ref 0.000^*^ 0.000^*^ 0.025^*^ 0.007^*^ 0.006^*^ 0.002^*^	- 49.486 29.963 4.455 5.773 5.945 7.484	- 10.920–224.253 7.379–121.674 1.210–16.410 1.626–20.497 1.655–21.357 2.091–26.780
**Water source**					
Turaa Canar Barraled	95 30 93	33 (34.7) 24 (80.0) 53 (57.0)	Ref 0.001^*^ 0.000^*^	2.778 8.113	1.512–5.104 2.947–22.334

* *p* < 0.05 is significantly different

**Table 4:** Total herd levels of infection with CBPP in cattle in El Jazeera State

Locality	No of herd	No positive	Percentage positive (%)
El Manageel	3	2	67
Wad Madani	4	4	100
Eastern El Jazeera	3	2	67
El Kamleen	3	2	67
El Hasaheesa	3	2	67
Twenty-four El Gorashi	1	1	100
Southern El Jazeera	3	3	100
**Total**	**20**	**16**	**80**

## Discussion

Contagious bovine pleuropneumonia (CBPP) is an infectious respiratory transboundary disease affecting cattle. The disease has serious socioeconomic consequences at the farmer and national levels, hamping export potential. The overall seroprevalence of CBPP infection reported herein (50.5%) in cattle in El Jazeera State, Sudan, is higher than that reported in Khartoum State and Eastern states of Sudan by different investigators [17, 18, 20]. However, it is lower than the reported 57% in Khartoum State by Shareef [21] and about same as the reported 51.1% in the same state by Karam [22] (51.1%). CBPP surveillance is not been carried out in many African countries due to fragmented veterinary services and limited resources. Anyway, in Africa, lower CBPP seroprevalence has been reported in Angola (17.5%) [23], Ethiopia (8.1%) [24] and Niger (4.15%) [25]. The difference in seroprevalence rates may be due to the agro-ecological diversity of the study areas, animal management, population density, production system, and the applied technique to evaluate seroprevalence [24, 26].

The CBPP-specific antibodies revealed among cattle proposed natural infection, as CBPP vaccination was not applied in the study area. Furthermore, the screened animals were aged > 6 month; thus, maternal immunity was no longer existing.

Comparative studies between the CFT and ELISA techniques conducted earlier have shown that the ELISA techniques are more sensitive than CFT in detecting CBPP antibodies in serum with very low titers, however, are less specific than the CFT [27, 28]. Marobela-Raborokgwe et al. [10] reported that there was 85% agreement between immunoblotting test (IBT) and c-ELISA and 80% agreement between c-ELISA and CFT.

In Sudan, vaccination against CBPP has been practised since 1944 using infected pleural exudates from sick animals. Later on broth culture vaccine using F strain, which isolated from Darfur province was applied [29]. At present, only one live attenuated freeze-dried is produced for CBPP vaccination [30].

In the present study, certain factors may have affected the seroepidemiology of CBPP infection in El Jazeera State, Sudan. Our study indicates that CBPP seropositivity was higher in females than in males. This is in line with the results obtained by Eslah et al. [18] in Khartoum State during 2016–2017. This may be due to the high number of screened female (*n* = 175) samples compared to the male (*n* = 43) samples examined in our study.

A significant association (*p* < 0.05) was also observed between CBPP seropositivity and cattle samples from different localities, as animals sampled from Eastern and Southern El Jazeera and Wad Madani localities were more likely to be infected. Teklue et al. [31] and Mamo [24] reported a significant association between the seroprevalence of CBPP and different agro-ecological systems in Ethiopia. Sudan has diverse agro-climatic conditions and almost all animals are owned by smallholder farmers or traditional pastoralists. El Jazeera State is located in semi-arid zone with three distinct seasons: winter (November to February), summer (April and May), and autumn (July to September), while March, June, and October are transitional months. Mobile pastoralism is predominant in this area, therefore during this movement the animals share common water and pasture route enabling the transmission of CBPP from one area to another.

Our study showed that water source for drinking is another potential risk factor that affects CBPP seropositivity in El Jazeera State; hence, cattle that drink from the canal were more significantly infected than cattle that drink from other water sources. This may indicate that canal as water source can facilitate the spread of CBPP through mixing of infected and susceptible animals besides while less common, the bacteria causing CBPP can survive for short periods on shared water sources. This may increase CBPP transmission and consequently the prevalence rate of infection.

CBPP antibodies were prevalent both at herd at individual animal level. The highest infection rate 100% among herds was observed in Southern El Jazeera, Wad Madani and Twenty four Gorashi is in concordance with high death reported from farms in this particular area (Hussien, unpublished data) due to CBPP, which was confirmed by marbled appearance of lung at postmortem examination.

## Conclusions

It was concluded that CBPP antibodies are prevalent in El Jazeera State, Sudan. The origin of the sampled cattle, sex and water source had a significant effect on the seropositivity for CBPP infection. Further surveillance of CBPP at the country level is important for estimating its economic impact on the animal industry in Sudan. In addition, major awareness both in the production area and quarantine centers is important, as CBPP appearance may result in restrictions on the trade of animals and animal products internationally. Effective vaccination and movement control are the best choices for disease control.

### Electronic supplementary material

Below is the link to the electronic supplementary material.

Supplementary Material 1

## Data Availability

Data and materials are available upon request by the corresponding author.

## Abbreviations

BTV: Bluetongue virus
cELISA: Competitive enzyme-linked immunosorbent assay
IgG: Immunoglobulin G
OIE: World organization for animal health
